# Selective CNS Targeting and Distribution with a Refined Region-Specific Intranasal Delivery Technique via the Olfactory Mucosa

**DOI:** 10.3390/pharmaceutics13111904

**Published:** 2021-11-10

**Authors:** Frank Maigler, Simone Ladel, Johannes Flamm, Stella Gänger, Barbara Kurpiers, Stefanie Kiderlen, Ronja Völk, Carmen Hamp, Sunniva Hartung, Sebastian Spiegel, Arghavan Soleimanizadeh, Katharina Eberle, Rebecca Hermann, Lukas Krainer, Claudia Pitzer, Katharina Schindowski

**Affiliations:** 1 Institute of Applied Biotechnology, University of Applied Science Biberach, Hubertus-Liebrecht Straße 35, 88400 Biberach, Germany; maigler@hochschule-bc.de (F.M.); ladel@hochschule-bc.de (S.L.); flamm@hochschule-bc.de (J.F.); gaenger@hochschule-bc.de (S.G.); voelk.ronja@web.de (R.V.); carmen.mirjam.hamp@t-online.de (C.H.); sunnivahartung@web.de (S.H.); spiegel@hochschule-bc.de (S.S.); soleimani@hochschule-bc.de (A.S.); kathy-eberle@web.de (K.E.); rebecca.hermann96@gmail.com (R.H.); 2 Faculty of Natural Science, University of Ulm, Albert-Einstein-Allee 11, 89081 Ulm, Germany; 3 Medical Faculty, University of Ulm, Albert-Einstein-Allee 11, 89081 Ulm, Germany; 4 Interdisciplinary Neurobehavioral Core, Heidelberg University, Im Neuenheimer Feld 515, 69120 Heidelberg, Germany; barbara.kurpiers@pharma.uni-heidelberg.de (B.K.); claudia.pitzer@pharma.uni-heidelberg.de (C.P.); 5 Prospective Instruments LK OG, Stadtstraße 33, 6850 Dornbirn, Austria; sk@p-inst.com (S.K.); lk@p-inst.com (L.K.)

**Keywords:** CNS drug delivery, nose to brain drug delivery, biopharmaceuticals, therapeutic antibodies, CNS targeting, refined drug delivery

## Abstract

Intranasal drug delivery is a promising approach for the delivery of drugs to the CNS, but too heterogenous, unprecise delivery methods without standardization decrease the quality of many studies in rodents. Thus, the lack of a precise and region-specific application technique for mice is a major drawback. In this study, a previously developed catheter-based refined technique was validated against the conventional pipette-based method and used to specifically reach the olfactory or the respiratory nasal regions. This study successfully demonstrated region-specific administration at the olfactory mucosa resulting in over 20% of the administered fluorescein dose in the olfactory bulbs, and no peripheral bioactivity of insulin detemir and Fc-dependent uptake of two murine IgG1 (11C7 and P3X) along the olfactory pathway to cortex and hippocampus. An scFv of 11C7 showed hardly any uptake to the CNS. Elimination was dependent on the presence of the IgG’s antigen. In summary, it was successfully demonstrated that region-specific intranasal administration via the olfactory region resulted in improved brain targeting and reduced peripheral targeting in mice. The data are discussed with regard to their clinical potential.

## 1. Introduction

With growing numbers of patients suffering from neurological disorders, the need arises for treatment and administration approaches with the ability to deliver drug molecules in therapeutic quantities to the central nervous system (CNS). Yet, this need is nearly unmet [1]. Drug delivery technologies are of high relevance as they ensure that a pharmaceutical compound can reach its pharmacological target, and thereby safely achieve its desired therapeutic effect [2]. A highly critical point in drug delivery is the low availability of drugs in the CNS due to the blood brain barrier (BBB). The purpose of the BBB is to protect the brain by restricting the entry of neurotoxic substances. This is achieved by the tightly composed endothelial barrier, a structure that represents a major hurdle for drug molecules to enter the CNS [3]. While for small molecule drugs, chemical modification may improve CNS delivery, nearly all macromolecules such as proteins and antibodies fail to cross the BBB in therapeutically relevant amounts [4,5]. Drugs with a low central bioavailability are currently delivered via intrathecal, intracerebroventricular or intraparenchymal injections. In this way, they are delivered directly to the cerebrospinal fluid (CSF) of the CNS. However, such invasive delivery systems are also accompanied with lower patient compliance and a documented risk of infections and side effects [6]. 

In recent past decades, the minimally-invasive approach of intranasal drug administration from the nose to the brain (N2B) has gained considerable attention as an alternative drug delivery route. The nasal cavity is highly suitable for minimally-invasive drug delivery, since the airway mucosa presents a good permeability and efficient absorption of both small molecule drugs and biopharmaceuticals [7]. Intranasal drug transport via neuronal connections such as the olfactory and trigeminal nerves appears to be the most relevant pathway to reach the CNS [8,9]. Moreover, the human nasal cavity has an impressively large mucosal surface area of about 160 cm^2^. Further advantages include the rapid drug uptake after administration and avoidance of the hepatic first-pass elimination [10]. Challenges of intranasal N2B drug administration are mainly represented by the mucociliary clearance, limited administrable volume, enzymatic degradation and physico-chemical properties of the mucus layer, such as low pH [11,12].

In more detail, the nasal mucosa is not uniform and consists of different epithelia [6]. The two predominant epithelial types are described as respiratory and olfactory epithelium, and most likely constitute the main areas of substance absorption. Nasal respiratory epithelium is formed by ciliated cells, goblet cells, intermediate cells, and basal cells. Its further constituents are the various serous glands that produce the nasal mucus and nasal secretions that are propelled from the ciliated cells to the nasopharynx [13,14]. The respiratory mucosa covers up to 90% of the nasal cavity in humans and up to 50% in rodents [12] and is highly perfused, hence very suitable for the systemic absorption of drugs [15]. 

The human olfactory cleft extends from the roof of the nasal cavity down to the superior parts of the turbinates and is covered with olfactory mucosa [16]. The olfactory mucosa covers about 50% of the nasal cavity in rodents and less than 10% in humans [17]. It is formed by pseudo stratified columnar epithelial cells, olfactory sensory neurons (OSN), supporting cells, basal cells and Bowman’s glands [15]. The unmyelinated axons of OSN spread through the basal lamina and form the *fila olfactoria* nerve bundles enclosed by olfactory ensheathing cells (OEC) and olfactory nerve fibroblasts. OECs are suggested to be a part of the innate immune system and their uptake of particles is well described [18,19]. The ensheathed nerve bundles travel through the cribriform plate of the ethmoid bone into the CNS where they terminate at the olfactory bulb. Hence, OSN are exceptional neurons that have their cell bodies located in a distal epithelium while their non-motile cilia processes extend into the mucus and allow them to be in direct contact with the environment. Interestingly, the olfactory nerve bundles appear to play a major role in N2B drug delivery. The intranasal delivery of drugs to the CNS has been pinpointed to the upper third of the nasal cavity, thus the olfactory region [4,6,12]. 

To reach the nerves, the drug must pass the epithelial tight junctions, which poses a hurdle that could limit the drug uptake [12]. However, the neuronal turnover within the olfactory epithelial layer is rather high, and dying cells may leave a gap that renders the epithelium sufficiently porous [20]. Although new OSN regrow in the gaps, the clefts may still allow an efficient drug uptake for larger particles. In addition, the formation of tight junctions lining the apical layer has been reported to be delayed [21]. Despite these observations, it should be noted that the passage through the *lamina propria* does not consequently imply that the total applied dose of the drug will arrive in the CNS. It may also be absorbed by blood vessels, enter glands, lymphatic/glymphatic vessels, the cranial nerves or interact with mucosal immune cells [6]. However, mathematical predictions strongly suggest a transport along the olfactory and trigeminal neural pathways [22]. Taking the trigeminal or olfactory route, the drug can reach the subarachnoid space adjacent to the pons or the olfactory bulb. From here, the further distribution of a drug in the CNS appears to be mediated via bulk flow of the CSF [23]. 

Owing to the promising features of intranasally delivered CNS-active drugs, there has been a rise in publications on intranasal drug delivery experiments in rodents. However, a literature search on in vivo experiments that use intranasal drug delivery has revealed one concerning condition. Nearly every intranasal administration approach is performed by pouring a drop of drug solution onto, or into, the nostril of a mouse or rat. In this scenario, even the administration of volumes of 20 µL, or less, lead to a flooding of the murine nostril, so that the total amount of deposited drug remains unknown. Furthermore, this approach is also accompanied with safety issues for the laboratory animals such as the swallowing or inhalation of administered drug solution. Further, the assessment of pharmacokinetic data and of quantitative readouts cannot be determined when using this conventional technique [24]. 

**Figure 1:**
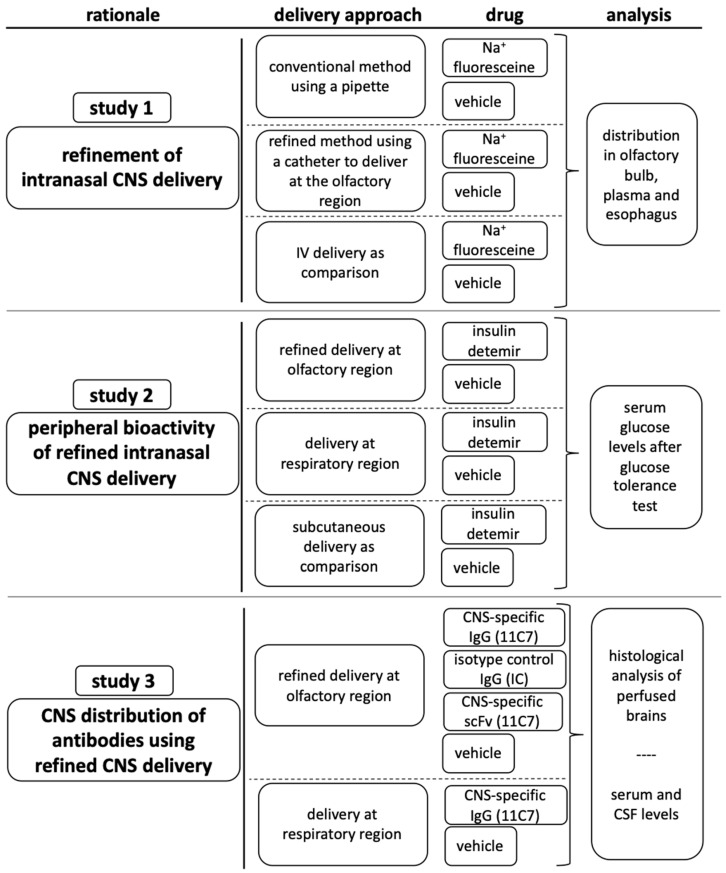
Study design of three studies with the aim of validating the previously refined intranasal region-specific administration technique [25] in vivo at the olfactory mucosa (study 1), determining the extent of molecules that are delivered to the periphery when administered at the olfactory mucosa (study 2) and analyzing the distribution of IgGs in the CNS after intranasal administration at the olfactory region (study 3).

Recently, the authors of this study have suggested a region-specific administration approach utilizing a catheter to either target the respiratory or olfactory mucosa [25]. This approach was developed with the aid of a 3D cast of the murine nasal cavity. As the flow chart in Figure 1 summarizes, the conventional and refined region-specific intranasal administration were first compared to examine the novel administration approach in more detail and to verify its suitability and feasibility. Then, the potential uptake to the periphery after intranasal delivery was explored. Here, insulin was used as it is well described with the conventional intranasal method [26] and as it provides peripheral bioactivity. Finally, the time-dependent distribution of modern biopharmaceutics within the CNS was evaluated using the novel intranasal technique. In accordance with former investigations, different molecular sizes were compared [27]. In summary, the data indicated that this novel refined intranasal technique is highly suitable for further studies to understand the N2B transport mechanisms and to explore the pharmacokinetics of complex biopharmaceutics after region-specific intranasal delivery.

## 2. Materials and Methods

Unless stated elsewhere, all chemicals were purchased from Sigma Aldrich, Taufkirchen, Germany.

### 2.1. Manufacturing of the Antibodies 11C7, P3X (Isotype Control) and 11C7 scFv

The hybridoma cell line 11C7 producing a murine IgG1 directed against Nogo-A was kindly provided by Prof. Dr. Martin Schwab/NovaGo Therapeutics AG (Schlieren, Switzerland). Hybridoma cells were cultivated on an orbital shaker (Kuhner, Herzogenrath, Germany) at 140 rpm and 37 °C in a humidified atmosphere with 5% CO_2_ within serum-free TurboDoma TP-6 medium (Cell Culture Technologies LLC, Gravesano, Switzerland) supplemented with 0.1% (*v*/*v*) Pluronic F-68 (Merck, Darmstadt, Germany) and 4 mM L-Glutamine (Lonza, Basel, Switzerland). Batch production processes were performed within 1000 mL disposable Erlenmeyer shake flasks with vented caps (Corning, New York City, NY, USA) and a working volume of 350 mL with an initial seeding density of 4 × 10^5^ cells/mL. A murine IgG1 was used as non-binding isotype control and produced with the hybridoma cell line P3X63Ag8 (ATCC, Manassas, VA, USA). Cells were cultivated under static conditions at 37 °C in a humidified atmosphere with 5% CO_2_ within T175 flasks with vented caps (Greiner Bio-one, Frickenhausen, Germany). Cultivation and production were performed in Dulbecco’s modified Eagles’s medium supplemented with 4 mM L-Glutamine and 10% (*v*/*v*) XerumFree^TM^ (Amsbio, Cambridge, MA, USA) with a working volume of 40 mL and an initial seeding density of 5 × 10^5^ cells/mL. Cultivation parameters for both cell lines were assessed daily and the harvesting procedure was initiated prior to when the viability declined to ~70%.

Prior purification, cell culture broth was cleared by centrifugation at 3000× *g* for 15 min and subsequent microfiltration by using a Sartopore^®^ 2 capsule (Sartorius, Göttingen, Germany) with a heterogeneous PES double layer. The purification was performed using a ProteinA MabSelect SuRe™ resin (GE Healthcare, Solingen, Germany) packed into a XK 16/40 column (GE Healthcare, Solingen, Germany) and an ÄKTA Purifier system (GE Healthcare, Solingen, Germany). After finishing the clearing procedure, culture broth was loaded on the pre-equilibrated resin, which was performed with 10 mM phosphate buffer and 140 mM NaCl pH 7.0. To remove unbound material, the resin was washed with the equilibration buffer and finally antibody was eluted using 20 mM sodium acetate buffer with pH 3.0. Subsequently, elution fractions were pooled and introduced to diafiltration via tangential flow filtration using a Sartocon^®^ slice 200 stainless steel holder (Sartorius, Göttingen, Germany), equipped with a 30 kDa molecular weight cut-off Hydrosart^®^ ultrafiltration cassette (Sartorius, Göttingen, Germany). Diafiltration of both antibody solutions was performed against phosphate buffered saline (PBS) at pH 6.5 with concomitant concentration up to a final concentration of 10 mg/mL. Continuous determination of antibody concentrations was facilitated by using a NanoDrop™ (Thermo Fisher Scientific, Dreieich, Germany) spectrophotometer at 280 nm and applying the mass extinction coefficient 13.7 L/(g·cm). 

The recombinant His-tagged 11C7 scFv (approximately 31 kDa) was produced in a stable and monoclonal CHO-K1 cell line by batch cultivation in 1000 mL shake flasks (Omnilab, Munich, Germany) under orbital shaking conditions (50 mm orbit) at 140 rpm, 37 °C within a moist saturated atmosphere and 5% CO_2_ saturation. With an initial seeding density of 4 × 10^5^ cells/mL, 400 mL HyClone SFM4CHO (Cytiva, Freiburg, Germany) supplemented with 4 mM L-Glutamine and 500 µg/mL G418 were inoculated and cultivated until the viability declined to ~70%. Cell culture broth was cleared by centrifugation at 3000× *g* for 15 min and subsequent microfiltration by using a Sartopore^®^ 2 capsule (Sartorius, Göttingen, Germany) with a heterogeneous PES double layer. The purification was performed using a HisTrap™ FF 5 mL prepacked column (GE Healthcare, Solingen, Germany) and an ÄKTA Purifier system (GE Healthcare, Solingen, Germany). The cleared culture broth was loaded onto preequilibrated resin (equilibration buffer: 20 mM tris (hydroxymethyl) aminomethane, 300 mM NaCl, 10 mM imidazole, pH 8.0). To remove unbound material, a washing step with equilibration buffer and a total of 20 mM imidazole was performed. Elution of bound 11C7 scFv was initiated by gradually increasing imidazole concentration to 500 mM. Subsequently, elution fractions were pooled and introduced to diafiltration via tangential flow filtration using a Sartocon^®^ slice 200 stainless steel holder (Sartorius, Göttingen, Germany), equipped with a 10 kDa molecular weight cut-off Hydrosart^®^ ultrafiltration cassette (Sartorius, Göttingen, Germany). Diafiltration of 11C7 scFv was performed against phosphate buffered saline (PBS) at pH 6.5 with concomitant concentration up to a final concentration of 2.4 mg/mL. Continuous determination of antibody fragment concentration was facilitated by using a NanoDrop™ (Thermo Fisher Scientific, Dreieich, Germany) spectrophotometer at 280 nm and applying the mass extinction coefficient 3.4 L/(g·cm).

### 2.2. Animal Housing and Ethics

Breeding pairs from male and female C57BL/6N mice were purchased from Charles River Laboratories (Sulzfeld, Germany) at the age of 8 weeks. Mice were expanded for one generation at the Interdisciplinary Neurobehavioral Core (INBC), University of Heidelberg. Male mice at the age of 11 weeks were used for the study. Mice were housed in groups of four per cage, with food and water ad libitum under a standard 12 h light/dark cycle (7:00 p.m.–7:00 a.m.) with a regulated ambient temperature of 22 °C and at a relative humidity of 40–50%. All procedures were conducted in strict compliance with national and international guidelines for the Care and Use of Laboratory Animals. Animal experiments were approved by the local governing body (Regierungspräsidium, Karlsruhe, Germany, under the ethical approval number G-92/19) and were carried out in compliance with the ARRIVE guidelines.

### 2.3. Study 1: Refined Region-Specific Catheter-Based Intranasal Administration of Sodium Fluorescein

A total number of 24 C57BI/6 mice were randomized to receive vehicle (PBS) or sodium fluorescein (10 mg/mL in PBS; Sigma Aldrich, Taufkirchen, Germany) solution administered either via the conventional or refined intranasal application or via intravenous (IV) injections. For the conventional application, animals were anesthetized with Isoflurane and placed in a supine position, with the head supported at a 45 degree angle to the body. Volumes of 2 µL of either vehicle or sodium fluorescein were applied to the nostril and repeated with the other nostril. The refined region-specific administration was established with 3D cast reproduced from a CT scan of murine skull [25]. Animals were anesthetized with Isoflurane and placed in a supine position, with the head supported at a 45 degree angle to the body. Volumes of 2 µL were applied to one ethmoid turbinate through the nostrils using a neonatal catheter (Nutriline, Vygon GmbH und Co KG, Aachen, Germany) with a Hamilton 10 µL-syringe (VWR International GmbH, Darmstadt, Germany). For an irritation-free application, a nasal balm (Bepanthen Augen- und Nasensalbe, Bayer Vital GmbH, Leverkusen, Germany) was applied at the sides of the catheter. The catheter was inserted 8 mm ± 0.1 mm from the nostril and with an insertion angle of 60 degrees. Fifteen seconds after application, the catheter was slowly removed and the procedure was repeated with the other nostril. For IV application the dose was administered via the tail vein according to standard procedures for mice. Samples (blood) and organs (brain, olfactory bulbs, esophagus, stomach) were collected at 10 and 30 min after administration. Organs were minced with chilled PBS (pH 7.4) in a Potter–Elvehjem homogenizer and centrifuged. The supernatant was transferred to a 96 well plate (Greiner Bio-One Fluotrac) and analyzed in a fluorescence plate reader with λ_ex_ 460 nm and λ_em_ 515 nm (SpectraMax M Series Multi-Mode Microplate Reader, Molecular Devices, San Jose, CA, USA). Quantification was performed with serial dilution of sodium fluorescein in the respective organ matrix. Blood samples were collected in tubes, (S-Monovette Sarstedt, Nümbrecht, Germany), centrifuged, and the plasma samples were analyzed as described above.

### 2.4. Study 2: Region-Specific Administration of Insulin Detemir (Levemir^®^)

Animals were randomized in groups to receive either saline (0.9% NaCl saline, Miniplasco connect, B.Braun AG, Melsungen, Germany) or insulin detemir (Levemir^®^, NovoNordisk, Denmark) with the refined technique at the respiratory or olfactory region. Blood samples were collected from the saphenous vein 5 min prior to dosing and baseline blood glucose levels were determined with Contour^®^ blood glucose test strips (Ascensia Diabetes Care, Parsippany, NJ, USA). Region-specific administration at the olfactory area was performed as stated above (study 1) by introducing the catheter 8 mm into the nostril and applying 2.5 µL per nostril. To target the respiratory region, the catheter was introduced only 2 mm. Blood glucose levels were determined 10 min after the intranasal delivery. Animals were challenged with a glucose tolerance test by intraperitoneal administration of 3 g/kg glucose 15 min after intranasal delivery and blood glucose levels were determined 30 and 75 min after intranasal dosing. 

### 2.5. Study 3: CNS Distribution of Monoclonal Antibodies and a scFv after Region-Specific Administration

Application procedures at the olfactory and respiratory region were performed with 3 µL per nostril as described above. Amounts of 11C7 and P3X as isotype control were formulated in PBS pH 6.5 at 10 mg/mL and 11C7 scFv at 2.4 mg/mL to provide equimolar doses. Animals were anesthetized, CSF was collected from the cisterna magna and finally mice were transcardially perfused with saline. CSF samples were stored at −20 °C until analysis. The skin of the mouse heads was removed and the heads stored in 4% paraformaldehyde until further processing. Mice heads were dehydrated with sucrose and 14 µm sagittal cryosections were cut at −25 °C from the nostril through the cerebellum (cryostat HM525 NX Thermo Fisher Scientific, Dreieich, Germany) and dried at 60 °C (UFB400, Memmert GmbH & Co KG, Schwabach, Germany).

### 2.6. Staining Procedures and Microscopy

Thawed sections were washed three times with PBS (5 min, 10 min and 15 min) and blocked with blocking buffer (PBS, 4% fetal bovine serum, 0.5% Triton X-100 and 5% normal goat serum) for 3 h. For the detection of 11C7 full IgG and P3X, AF647 alpaca anti-mouse (#615-605-214, Jackson Immuno Research, Cambridgeshire, UK) was diluted 1:500 in PBS and incubated for 2.5 h at room temperature. Following three washing steps, sections were counterstained with 20 µg/mL DAPI (4’,6-diamidin-2-phenylinol; Sigma Aldrich, Taufkirchen, Germany) in PBS for 10 min. Slides were washed with PBS for 10 min, followed by washing with water for 1 min and mounted in Fluoromount G (Thermo Fisher Scientific, Dreieich, Germany). The images were captured with a confocal laser microscope (LSM 710, Zeiss, Jena, Germany) at the Core Facility Confocal and Multiphoton Microscopy at Ulm University, Germany, and with the MPX-1040 multi-modal microscope (Prospective Instruments, Dornbirn, Austria) for whole slide images. Whole slide images were acquired using a 4× (Thorlabs, Super Apochromatic, Air, NA 0.2) and a 16× (Nikon LWD Plan Fluorite, Water, NA 0.8) objective at three excitation channels: blue (λ_ex/em_ 395/432 nm), green (λ_ex/em_ 475/515 nm) and red (λ_ex/em_ 637/681 nm). Whole slide images were scanned using an xyz-stage and an in-house developed software routine (Prospective Instruments, Dornbirn, Austria). All images were saved as TIF-files and postprocessed using ImageJ (v1.53c) as previously described [28].

### 2.7. Statistics

Data were assessed for statistical significance using Prism Version 8.3.0 (GraphPad, San Diego, CA, USA). All data are presented as mean ± SEM. Statistical significance was calculated with ANOVA or Student’s *t*-test as indicated.

## 3. Results

### 3.1. Refinement of Intranasal Delivery and Establishing a Region-Specific Administration at the Olfactory Region (Study 1)

**Figure 2:**
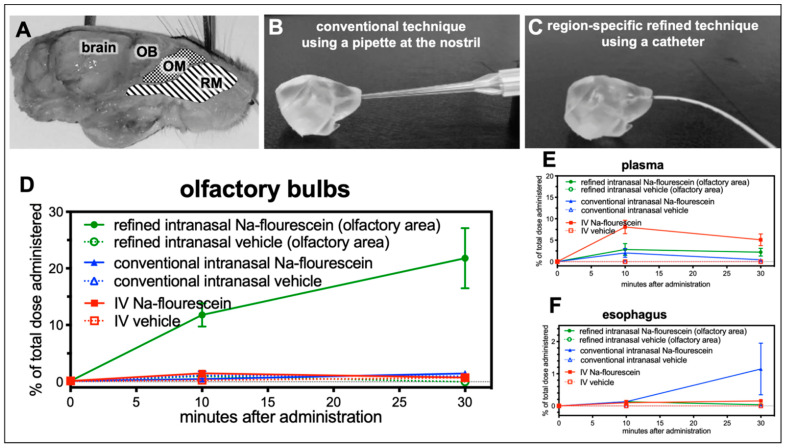
The refined technique results in up to 21% of the dose in the olfactory bulbs, when administered at the olfactory region (study 1). (**A**) Sagittal view on a murine head with highlighted areas for catheter-based administration at either respiratory mucosa (RM) or olfactory mucosa (OM) with adjacent olfactory bulb (OB) as part of the brain. As previously described, a region-specific technique was developed with the aid of 3D-printed models of the murine nasal cavity reconstructed from CT scans of a mouse head [25]. (**B**) demonstrates the conventional technique using a pipette with usually a rather high volume, from 20 to 30 µL. With this method, no selective delivery to the respiratory or olfactory mucosa is possible. Therefore, the intranasal delivery (**C**) has been refined by using a catheter with an adjusted smaller administered volume, which can deliver the drug region-specific either at the olfactory or respiratory area. The aim of study 1 was to validate the region-specificity of the refined catheter-based technique with fluorescein in vivo. (**D**) 10 min after administration, the levels reached with the refined technique were significantly higher than achieved with the conventional method or IV delivery (* *p* < 0.04, Student’s *t*-test). At 30 min after administration at the olfactory region, 21.8 ± 5.34% of the dose was recovered at the olfactory bulbs while administration with a pipette at the nostrils resulted in only 1.5 ± 0.05% (n = 2; *p* = 0.0626) and intravenous (IV) delivery resulted in 0.7 ± 0.07% (n = 2; *p* = 0.0587). (**E**) Elevated levels of fluorescein were detected in serum after IV administration and after intranasal delivery with the conventional pipette-based method. (**F**) Interestingly, higher levels of traces of the intranasally administered doses were swallowed and ingested with the conventional method than with the refined method (conventional: 1.15 ± 0.804% vs. refined: 0.03 ± 0.078% vs. IV: 0.078 ± 0.078%). Due to the limited number of animals per group this study shows tendencies, but fails to demonstrate statistical significance (mean ± SEM; n = 2). (**A**–**C**) are adapted with permission from ref. [25]. 2021, Nicole Lange et al.

The nasal cavity is covered by different epithelia and different nerve endings such as the olfactory and the trigeminal nerve, which are embedded in the mucosa at different anatomical regions (Figure 2A). In order to gain a profound understanding of the fate and pathways of intranasally administered molecules, a region-specific administration technique enabling the discrimination between different nasal epithelia is of high importance The conventional technique to deliver drugs intranasally is to administer drops to the nostrils with the aid of a pipette (Figure 2B). Despite the fact that in most published studies the administered volumes exceed the volume of a murine nasal cavity, this method is not suitable to target only one region covered predominantly with a specific epithelium such as olfactory or respiratory mucosa (Figure 2A). Lochhead and Thorne suggested a potential pathway for drug delivery into the CNS via the olfactory mucosa [12]. Therefore, the authors of this study recently developed a novel catheter-based technique in vitro to target the olfactory or respiratory region only, with a smaller volume and with high specificity and reproducibility (Figure 2C) [25].

Here, this refined technique was evaluated for the first time in vivo in a small pilot study (study 1; Figure 1). A solution containing 20 µg of sodium fluorescein or vehicle (PBS) was administered either to the nostril (conventional technique) or via a catheter to the olfactory area (refined technique). Although, volumes of 20 to 40 µL per nostril had been reported for the conventional technique, here the same volume as for the refined technique was used to enable a direct comparison. Finally, a group of animals received fluorescein or vehicle intravenously (IV) into the tail vein. Due to the rather fast elimination of fluorescein, animals were sacrificed 10 and 30 min after application and the fluorescence was determined in olfactory bulbs, whole brains (without olfactory bulbs), serum and esophagus. No relevant increase in fluorescence levels was found in any sample from animals which have received vehicle (data not shown). As demonstrated in Figure 2D significant levels of sodium fluorescein were detected with the refined technique in olfactory bulbs (11.76 ± 2.05% of the total administered dose after 10 min, and 21.81 ± 5.34% after 30 min) while the conventional method (0.50 ± 0.50% after 10 min, and 1.48 ± 0.05% after 30 min) and IV administration (1.49 ± 0.12% of the total administered dose after 10 min, and 0.74 ± 0.07% after 30 min) revealed only slightly elevated levels compared with vehicle. 

Levels in whole brains were marginally elevated up to 3.6% in the refined and IV groups after 10 min and in all three after 30 min (data not shown). As seen for olfactory bulbs, the levels in the IV group declined with time, apparently due to the short half-life of fluorescein. The similarly low levels of both intranasal groups may represent transport via the trigeminal nerve that has terminal endings in the respiratory and the olfactory region. In addition, the amount of fluorescein that could be detected in serum after both delivery techniques was determined. While 8.1 ± 1.57% were found in the serum 10 min after IV delivery, only 2.84 ± 1.38% (refined) and 2.04 ± 1.02% (conventional) could be detected. These values decreased after 30 min for the conventional technique and IV administration and remained rather stable with the refined technique (Figure 2E). 

A high risk of the conventional technique is that a significant amount of the delivered dose is swallowed and ingested or even inhaled and thereby life-threatening for the animals [25]. Therefore, esophagus and stomach were analyzed for potential ingested traces of fluorescein. Samples from the stomach could not be used since the animals had not been fasted for a sufficient time, but increased levels up to 1.15 ± 0.80% were found in animals treated with the conventional technique compared with refined (0.13%) and IV (0.16%; Figure 2F) delivery. It should be noted that only a small volume was used for the conventional technique, while in published studies 10 to 20 times higher volumes are used. Therefore, the percentage of swallowed and ingested drug solution can be estimated to be rather high. In summary, the present data demonstrate that the refined technique is a suitable method to investigate drug delivery via the olfactory region.

### 3.2. Peripheral Bioactivity after Region-Specific Intranasal Administration of Insulin Detemir (Study 2)

**Figure 3:**
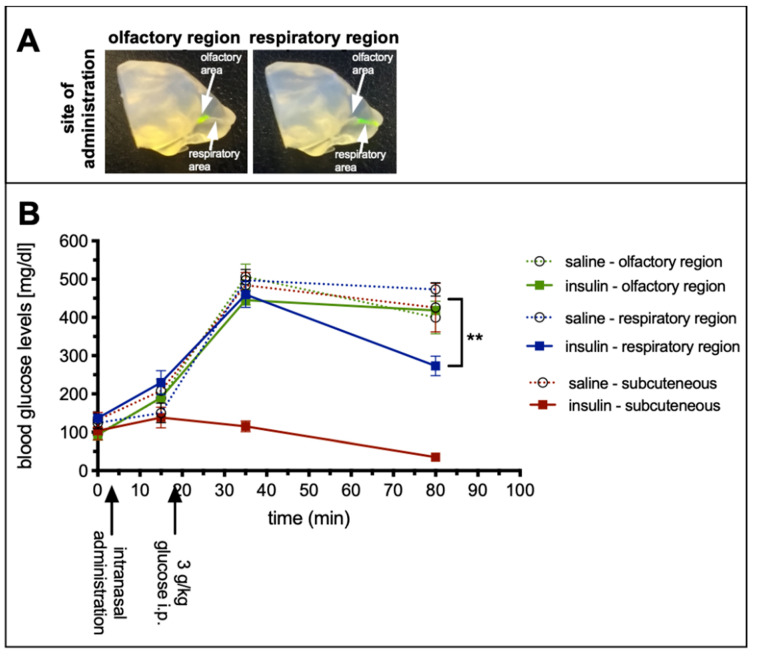
No peripheral bioactivity after region-specific delivery of insulin detemir to the olfactory region (study 2). Silicone based 3D-model made by vacuum cast method (**A**), for the visualization of the targeted regions [25]. Adapted with permission from ref. [25]. 2021, Nicole Lange et al. Glucose tolerance test to examine the peripheral effects after region-specific administration of insulin detemir versus vehicle (**B**), monitored via blood glucose levels. Black arrows highlight catheter-based refined administration to the respiratory or olfactory nasal regions or subcutaneous injections of insulin detemir or vehicle. Mice were challenged with a high dose of intraperitoneal glucose to determine the peripheral activity of insulin detemir. As expected, subcutaneously delivered insulin demonstrated a high peripheral bioactivity by lowering the blood glucose levels. Interestingly, insulin delivered via the respiratory regions covered predominantly with respiratory mucosa was also distributed from the nose to the periphery and displayed 40.2% of the subcutaneously delivered activity. Insulin targeted to the olfactory regions did not show any statistically significant bioactivity in the periphery. Error bars represent mean ± SEM, n = 3. Data were analyzed with two-way ANOVA with multiple comparisons. ** *p* < 0.009. A is reproduced from [25] with kind permission from the rights owner.

In general, mucosal tissues are well perfused and harbor several capillaries and blood vessels in their *lamina propria*. However, it is known that respiratory mucosa at the lower parts of the nasal cavity contains more and larger glands and more cavernous bodies than the olfactory mucosa [15]. Therefore, the aim of study 2 was to determine the peripheral bioactivity of a drug, which was administered at the olfactory or respiratory regions of the nasal cavity. Insulin was chosen as the drug since its peripheral bioactivity (lowering blood glucose levels) can be sensitively and easily assessed. Since the half-life of insulin is rather short, insulin detemir with a prolonged half-life was used [29]. Hence, mice were challenged with a high dose of glucose to uncover the amounts of insulin that was distributed to the periphery. As positive control, insulin detemir was administered subcutaneously (SC). As demonstrated previously, by introducing the catheter with the refined technique either 2 or 8 mm from the nostrils, different regions of the nasal cavity were targeted (Figure 3A) [25]. 

Blood samples were taken from the saphenous vein prior to catheter-based intranasal delivery to the respiratory or the olfactory region via both nostrils or SC delivery with a total dose of 0.5 IU = 71 µg insulin detemir). Saline was used as vehicle control. The basal blood glucose levels of all groups were comparable (Figure 3B) and also 10 min after intranasal or SC delivery no statistically significant changes were observed. Fifteen minutes after drug delivery all animals were challenged with 3 g/kg glucose injected intraperitoneally (IP) and subsequently bled after 30 and 75 min. While the glucose levels of the SC insulin detemir group remained stable, all other groups, regardless of whether vehicle or drug, displayed a strong and significant increase in blood glucose. However, 75 min after drug delivery, the blood glucose concentration of the animals, which received the insulin via the respiratory area displayed significantly lower levels compared with vehicle control (273 ± 25 mg/dL vs. 433 ± 25 mg/dL; ** *p* < 0.009), while the levels from the olfactory targeted group were unaffected with 418 ± 10 mg/dL (*p* = 0.86; Figure 3B). The lowered glucose level of the respiratory targeted groups corresponded to 40.2% of the peripheral bioactivity of the 0.5 IU insulin detemir administered SC (3.8% for olfactory targeting). All vehicle groups displayed similar values that did not differ significantly from each other. Therefore, all vehicle groups were pooled for statistical analysis.

These results imply that insulin detemir was absorbed from the respiratory nasal mucosa and distributed to the periphery to a significantly higher extent than when administered to the olfactory mucosa. In combination with the result from study 1 showing a specific targeting of the olfactory bulbs as parts of the CNS, the catheter-based refined method was highly specific for intranasal CNS targeting with low peripheral effects. 

### 3.3. Antibody CNS Distribution after Region-Specific Intranasal Administration (Study 3)

**Figure 4:**
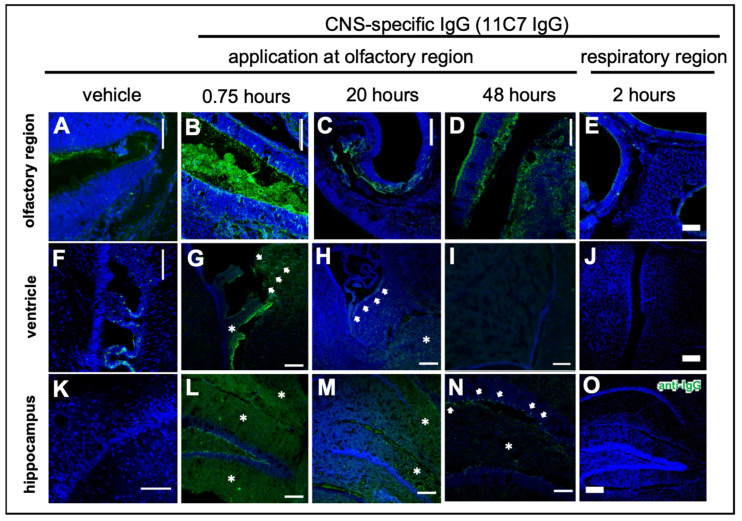
Distribution of the CNS-specific IgG1 antibody 11C7 in the nasal mucosa after region-specific intranasal delivery (study 3). A total dose of 60 µg 11C7 per mouse was administered at the olfactory (**B**–**D**,**G**–**I**,**L**–**N**) and respiratory (**E**,**J**,**O**) regions. The vehicle control was administered at the olfactory region and displays background staining from endogenous murine IgGs in the nasal mucus (**A**), and the choroid plexus (**F**), which was not removed after transcardial perfusion. The region-specificity became obvious in an investigation of the upper nasal cavity with its ethmoid turbinates: while after administration at the olfactory region the antibody was detectable at the olfactory mucosa (**B**), and showed a time-dependent clearance (**C**,**D**), no elevated levels of IgG were observed at the ethmoid turbinates after administration at the respiratory region. Further, none of the animals from the respiratory delivery group displayed any signs of a CNS delivery of 11C7 (**J**,**O**). A rapid distribution to the subventricular zones (see arrowheads) of the ventricles was found (**G**), that lasted up to 20 h (**H**), but was undetectable after 48 h (**I**). In addition, 11C7 was observed diffusely (see asterisks) in the hippocampus shortly after administration (**L**). The diffuse pattern disappeared within 20 h (**M**) and after 48 h distinct cells (see arrowheads) and neuronal projections were observed (**N**), highly similar to what was reported from Nogo-A expression studies [43]. Scale bar, 100 µm.

Modern biopharmaceutics such as antibodies and antibody-derived formats such as single chain variable fragments (scFv) have a very low bioavailability in the CNS when delivered intravenously [30]. Therefore, intranasal delivery to the CNS could be an attractive minimally invasive option. To explore the transport path from the olfactory region to the brain and to study kinetics of distribution and elimination, study 3 was designed with two different murine IgG antibodies and a scFv. The IgG1 antibody 11C7 binds to Nogo-A, which is predominantly expressed within the CNS but some reports have detected it in OSN [31,32]. The 11C7 was used as it is a promising antibody for multiple sclerosis or similar neurodegenerative diseases as it was shown that 11C7 therapy induces neuronal outgrowth in studies performed by Schwab and coworkers [33,34,35,36,37]. In general, the Fc part of IgGs is mentioned by other studies to be possibly involved in IgG uptake and transport either intra- or paracellularly [38,39,40]. However, there are conflicting data showing that the Fc binding neonatal Fc receptor (FcRn), which is the most prominent IgG transporter in mammals, functions mainly as an efflux transporter [40,41,42]. To investigate not only the impact of molecular size but also the presence of an Fc domain on molecular transport from the nose to the brain, the 11C7 N2B uptake was compared with an scFv from the same IgG. Furthermore, to exclude bias due to 11C7 binding potentially to Nogo-A on OSN, an isotype control antibody was used that does not bind to murine tissue. Therefore, the IgG1 antibody P3X was defined as the isotype control, since the absence of binding to murine brain, nasal mucosa or other investigated murine tissues in a dot blot (data not shown) could be confirmed. Both IgG antibodies were used at 10 mg/mL and the mice received 3 µL per nostril at the olfactory region using the catheter-based refined technique (total dose of 60 µg). The 11C7 scFv was delivered at equimolar concentrations with 2.4 mg/mL. PBS was delivered as vehicle control and one cohort received 11C7 at the respiratory region for comparison. Although 11C7 and P3X were biotinylated at the hinge region, detection was performed with polyclonal antibodies against murine IgG heavy and light chains since detection with streptavidin produced a rather high background. Mice were transcardially perfused in deep anesthesia, however, traces of endogenous murine IgGs could be found in particular in the nasal mucus and the choroid plexus (Figure 4A,F). Therefore, vehicle control showed baseline levels even if traces of endogenous IgGs were present.

The distribution in the nasal mucosa and to the CNS is shown in Figure 4 taken by confocal microscopy. In summary, the region-specificity was well demonstrated since no traces of 11C7 were found after administration at the respiratory region (Figure 4E) nor any distribution to the brain (Figure 4J,O). Compared with vehicle control levels, a rather rapid distribution to the subventricular zones and the hippocampus was observed. While in the beginning the staining pattern was rather diffuse (Figure 4G,L,M), within 48 h no 11C7 was detectable at the subventricular zones (Figure 4I) and a highly specific pattern remained in the hippocampus (Figure 4N) and cortex, similar to what was previously reported for Nogo-A expression, the antigen of 11C7 [43]. In addition, a similar rapid distribution to the olfactory bulb and midbrain followed by a fast elimination was recently observed with radiolabeled IgGs [44].

### 3.4. Fc-Dependent Uptake into Olfactory Mucosa and Transport along Neuronal Bundles to the CNS

**Figure 5:**
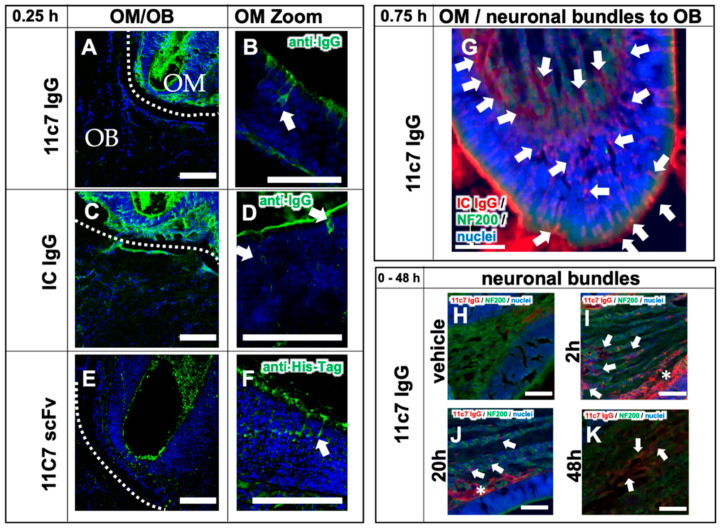
Transport mechanisms of both the full IgGs 11C7 and the isotype (IC) control, and the 11C7 scFv (study 3). Images from confocal microscopy show the rapid uptake of 11C7 and IC into the olfactory mucosa (OM) and also traces in the olfactory bulb (OB) after only 15 min (**A**,**C**). Higher magnification shows evidence for a predominantly intracellular pathway as previously reported from ex vivo olfactory mucosa (see arrowheads) (**B**,**D**) [38,39]. In contrast, the 11C7 scFv devoid of an Fc domain appears to be taken up to a lower extent (**E**) and displays more evidence for a transcellular transport (**F**). However, it should be noted that IgGs and scFv were visualized with different detections systems (anti-IgG vs. anti-His-Tag) and, hence, the fluorescence intensity should be evaluated with caution. Epiflourescence microscopy demonstrates, 45 min after administration, the full transport scheme with intracellular uptake at the apical mucosa, distribution to the *lamina propria* and transport along neuronal bundles from the *lamina* propria to the olfactory bulb (**G**). For a better visualization, anti-IgG immunoreactivity is displayed here in red, and neurofilament (NF200)-immunoreactivity in green. Transport kinetics along neuronal bundles after a single dose (**H**–**K**): while only background is observed in the vehicle control animals (**H**), decreasing levels of 11C7 are observed within 48 h (**I**–**K**). Arrowheads point to distinct stained structures while asterisk show diffuse staining pattern. Scale bar, 100 µm.

Recently, the transport of exogenously administered IgGs along neuronal bundles projecting from olfactory sensory neurons of the olfactory mucosa to the olfactory bulb in an ex vivo model of porcine olfactory mucosa [38] has been described. Excitingly, the Fc domain of IgGs appeared to play a major role in the transmucosal transport. Therefore, in study 3 the focus was also on the in vivo transport mechanisms from the olfactory region to the CNS (Figure 5) and the difference of full IgGs and a scFv devoid of Fc. After a single dose with 60 µg, a rapid uptake of both IgGs via intracellular pathways to the *lamina propria* (Figure 5A–D) and then along neuronal bundles to the brain, was observed (Figure 5G). As expected, the immunoreactivity of the IgGs along the neuronal projections decreased time dependently (Figure 5H–K). The 11C7 scFv with only 31 kDa and devoid of an Fc domain showed a distinct uptake pattern with evidence for an extracellular pathway and a decreased uptake (Figure 5E,F). Nevertheless, it should be noted that IgGs and scFv were detected with anti-IgG and anti-His-Tag antibodies, respectively. Therefore, no direct comparison of the staining intensity could be performed.

### 3.5. Reduced Elimination from CNS of Nogo-A-Binding IgG

**Figure 6:**
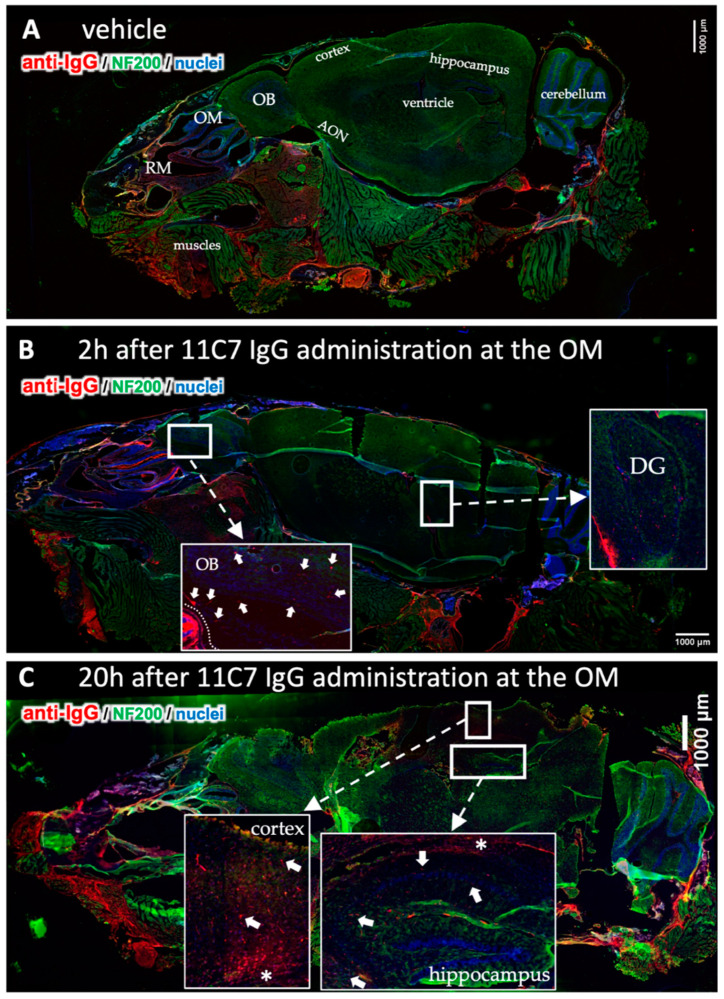
Time-dependent CNS distribution analyzed in sagittal section from mouse heads after region-specific delivery at the olfactory mucosa (OM, study 3). Whole slide images of a vehicle treated animal demonstrate endogenous IgGs in the nasal mucus, but also in the muscles underneath the nasal cavity and at the neck (**A**). This immunoreactivity was observed in all animals investigated. No background staining was observed in the CNS except for choroid plexus in all vehicle animals. The 11C7 could clearly be detected in the olfactory bulbs (arrowheads) and in the dentate gyrus of the hippocampus (**B**). At 20 h after administration the distribution pattern shifted into the caudal direction with lower intensities in the olfactory bulbs, but higher and more distinct staining in the cortex and the hippocampus where the antigen of 11C7 was expressed. Arrowheads point to distinct stained structures while asterisk show diffuse staining pattern. (**C**). OM, olfactory mucosa; RM, respiratory mucosa; OB, olfactory bulb; AON, anterior olfactory nucleus; DG, dentate gyrus. Scale bars, 1000 µm.

**Figure 7:**
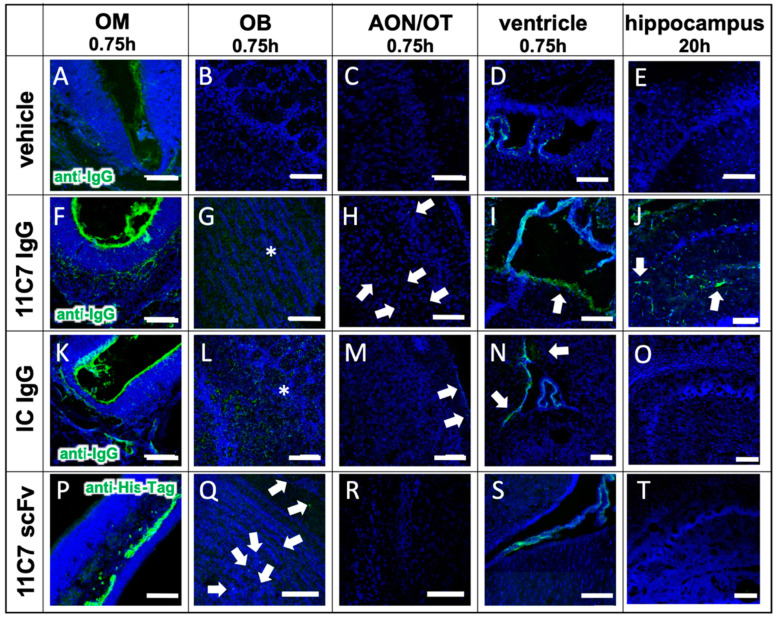
Antibody distribution is dependent on the presence of the antigen in the CNS and the presence of an Fc domain (study 3). The distribution and clearance in the murine OM and CNS are shown 45 min and 20 h (**E**,**J**,**O**,**T**) after directed intranasal administration at the olfactory region (OM). All images were captured with a confocal microscope. Arrowheads point to distinct stained structures while asterisk show diffuse staining pattern. (**A**–**E**) Background signal due to endogenous IgGs, that have not been cleared by transcardial perfusion, and autofluorescence in the tissue of an animal, which received a vehicle control (PBS). (**F**–**J**) distribution of anti-Nogo-A monoclonal full antibody 11C7. The 11C7 was detected in the OM (**F**), the OB (**G**), the AON/OT (anterior olfactory nucleus/olfactory tubercle) (**H**), the choroid plexus in the ventricles (**I**), and after 20 h in the hippocampus (**J**). The strongest signal was detected in the olfactory epithelium followed by the olfactory bulb and the choroid plexus. In the hippocampus, the signals were amplified for a better visualization. (**K**–**O**) Distribution of the isotype control (IC) antibody, which does not recognize any structure of the murine CNS as antigen. The distribution profile within 45 min was similar to 11C7 however the immunoreactivity in the AON/OT was lower in all animals investigated. The similar distribution pattern in OM, OB, AON and the subventricular zones imply the relevance of the Fc receptor system. The absence of IC in hippocampus (**O**) is a strong indication that the presence of the antigen is critical to avoid rapid elimination. (**P**–**T**) A scFv format of 11C7 was distributed to the OB to a lesser extent than the IgGs. In addition, hardly any signals higher than the vehicle control could be found in the AON/OT, choroid plexus, subventricular zones, nor in the Nogo-A expressing cells in the hippocampus. Nevertheless, since the 11C7 scFv was detected via its penta His-Tag, the intensities of (**A**–**O**) should not be directly compared with (**P**–**T**). Representative images are shown. OM, olfactory mucosa; OB, olfactory bulb; AON, anterior olfactory nucleus; OT, olfactory tubercle. Scale bar: 100 µm.

To investigate the CNS distribution after region-specific catheter-based administration at the olfactory mucosa, sagittal sections through a whole mouse head from the nostrils to the cerebellum were performed, and whole section images were captured with a multi-modal microscope (Figure 6). The vehicle control demonstrated the background immunoreactivity against murine IgGs (Figure 6A): No specific staining was observed within the CNS, but endogenous IgGs could be detected in the nasal mucus, in particular at the respiratory mucosa (RM). A rather high staining was observed in muscle tissue of the neck, jaw and cheeks in all animals investigated with a similar intensity (Figure 6A–C), however, this did not interfere with the analysis of the brain and nasal mucosa. In general, the intranasally administered antibody distributed through the brain in a rostral to caudal direction (Figure 6B,C and Figure 7) with more diffuse staining shortly after administration in the olfactory bulbs (Figure 7G,L), anterior olfactory nucleus (AON) or olfactory tubercle (OT), Figure 7H,M) in the subventricular zones (Figure 7I,N), while after several hours a more distinct staining pattern developed dependent on the antigen localization (Figure 6C). Both IgGs showed mostly similar levels in the rostral brain subsequent to administration, however, a majority of mice that received the IC antibody tended to display a somewhat stronger immunoreactivity in the olfactory bulbs compared with 11C7. The elimination of the full IgGs appeared to be strongly dependent on the presence of the antigen in the CNS. While 11C7 is an antibody with a high affinity against its CNS-specific antigen Nogo-A (in-house data revealed a K_D_ of 14 pM, data not shown), P3X served as isotype control (IC) with no antigen in the murine brain and nasal mucosa. Indeed, immunoreactivity against P3X is absent in both hippocampus (Figure 7O) and cortex (data not shown) up to 48 h after application while 11C7-immunorectivity is still detectable (Figure 4N, Figure 6C and Figure 7J). It can be estimated that different antibodies with other CNS-specific antigens would display different distribution patterns. In some sections, the staining pattern of 11C7 in hippocampus (Figure 7J) and cortex (data not shown) implicated its presence in blood vessel. This was probably due to 11C7 elimination from the CNS via FcRn uptake, a process that is, amongst others, localized in endothelial cells [45]. Interestingly, staining of blood vessels was not observed in animals treated with P3X, possibly due to the relative low amounts of P3X that reached these brain areas. In a previous study from Thorne and coworkers using radiolabeled IgGs administered via the conventional technique in rats, a similar distribution pattern in olfactory bulbs and midbrain was demonstrated [44,46]. While the study by Thorne et al. demonstrated the occurrence of IgG transport via the trigeminal nerve, no evidence for such transport was found in this study. Additionally, in the above-mentioned study, the radiolabeled IgGs appeared within 10 min in the blood in similar levels as in the olfactory bulbs after intranasal delivery with the conventional technique [44,46].

Interestingly, not only the antigen and its specific expression in the CNS played a role in the distribution and pharmacokinetics after intranasal delivery to the olfactory region, but also the presence of an Fc domain appeared to be an important factor. Hence, while the 11C7 scFv targeted the same antigen Nogo-A within the CNS, its uptake into the nasal mucosa and from there to the olfactory bulbs appeared to be significantly reduced when compared with both full IgGs (Figure 5E,F and Figure 7P–Q). As mentioned above, the detection of full IgG and scFv was not identical, so fluorescence intensities should be interpreted with caution, but also the localization within the epithelial layer of the olfactory mucosa differs dramatically (Figure 5F). In previous studies, it has already been speculated that the presence of the Fc domain plays a pivotal role in a successful mucosal uptake and these data confirm this [38,39].

It should be noted that CSF samples were also analyzed from the mice in study 3 by ELISA, but no significant amounts of 11C7, IC, or 11C7 scFv could be determined. It cannot be excluded that methodological issues interfered with the analysis since the samples had to be frozen for logistical reasons. Possibly, this may hint that the distribution to the CNS does not include drainage to the CSF or is rapidly cleared from the CSF [47,48,49]. Further studies are necessary to elucidate the involvement of the CSF, but also of the glymphatic system [50,51,52,53]. 

## 4. Discussion

Intranasal drug delivery is widely discussed as a promising approach to deliver drugs to the CNS. To use the great potential of intranasal N2B drug delivery, important aspects and challenges have to be tackled to advance it to clinical routine [54]. An impressive in-depth analysis of Koslovskaya and coworkers underlined the importance of quantitative pharmacokinetic and bioactivity data [24]. Although many studies of intranasal delivery have been published until 2014, only a small minority of 3% presents quantitative, robust data that are suitable for a potential clinical development. While several important high-quality intranasal studies have been published since 2014, the individual studies differ from each other due to the heterogeneity of non-standardized application methods, which limits their interpretation. The most common described intranasal technique is the pipette-based conventional application method (Figure 2B) that was used in a multitude of intranasal in vivo studies [8,55,56]. This conventional method is usually performed with a minimum volume of 20 μL per nostril in mice and a minimum volume of 40 µL in rats, although many variations have been also published. A volume of 20 µL substantially exceeds the volume of the murine nasal cavity. Even the stepwise application of said amount may not reduce the risk for much of the volume being swallowed. Notably, we have delivered conventially an amount as little as 2.5 µL per nostril (in mice with a body weight below 25 g) in Study 1, upon which appeared clear indications that the applied volume was swallowed (Figure 2F). To the authors’ knowledge, this is the first time that the ‘undesired fate’ of intranasally applied substances is reported. Additionally, to the authors’ knowledge there have been no reports published on the percentage of animals that were asphyxiated by intranasal delivery of high volumes using the conventional method. Rodents are obligate nose breathers [17] and therefore this is a highly relevant issue for both ethical reasons and biometric estimations. Since starting to use the refined technique in mice, no complications associated with the intranasal delivery have yet been experienced. All mice recovered well after catheter-based intranasal administration and displayed normal healthy behavior. To date, in all the authors’ intranasal studies, no deaths have occurred prior to sacrificing the mice at the scheduled time.

Apart from the excessive volume, a further issue with the conventional method is that it is impossible to target the respiratory or the olfactory mucosa alone. Lochhead and Thorne have already suggested different N2B transport routes: the olfactory pathway and the trigeminal pathway [12]. They could demonstrate the distribution of insulin or insulin-like growth factor-I within the CNS of rats after conventional intranasal administration with the involvement of both pathways [55]. In a more recent study, the percentage of drugs that reached the olfactory nerve (presumably via the olfactory mucosa) and the trigeminal nerve (presumably via the respiratory mucosa) was analyzed after the use of the conventional method in rats with radiolabeled IgGs. Here, a far higher radioactivity was detected in the trigeminal nerve than in the olfactory nerve [44]. With a region-specific technique such as the one demonstrated in study 2, the in-depth mechanisms of N2B transport can now be addressed. In study 1, it was successfully demonstrated that the previously presented refined intranasal administration technique [25] was able to deliver 20% of the dose of a fluorescent tracer to the olfactory bulb, and was thus facilitated by the olfactory pathway.

Insulin has well-known peripheral metabolic effects and lowers the blood glucose concentration, but it also plays an important central role in cognition, learning and memory [56,57,58,59]. Furthermore, dysregulation of insulin is involved in the pathogenesis of Alzheimer’s disease, where it is associated with lower levels of insulin in the CSF [60] and with amyloid-beta regulation [61,62,63,64]. The choice to use insulin for study 2 was influenced by the fact that a large number of animal and clinical intranasal studies have been performed with insulin (see [54] for review). For instance, radiolabeled insulin distributes widely throughout the mouse brain following conventional intranasal administration, with the highest levels detected in the trigeminal nerve and the olfactory bulbs [56]. Intranasal insulin delivered with a ‘conventional’ manual pump spray in a clinical study with healthy volunteers was rapidly delivered to the CSF [65]. In the context of the data from studies 1 and 2, this could be interpretated as no relevant traces of insulin reaching the olfactory region when using a manual pump spray in the human nostril. In fact, the use of an intranasal insulin spray developed as Nasulin™ for the needle-free regimen of diabetes, was stopped after disappointing results in blood glucose control during a Phase 2a proof-of-concept clinical trial [66]. It can be only speculated that the absorption of insulin was too heterogenous due to high variations in the anatomy of the human nasal cavity and the non-standardized administration at different nasal epithelia. 

Intranasal insulin also slowed the development of cognitive decline in different disease models [56] and improved learning and memory in wildtype mice [64]. Even clinical trials in Alzheimer’s patients have been conducted with intranasal insulin detemir administered with an atomizer targeting predominantly the lower parts of the nasal cavity such as the respiratory mucosa [67,68,69]. Unfortunately, neither the animal nor the clinical studies present meaningful data of blood glucose levels [54]. It remains a matter of speculation if, with region-specific intranasal delivery, these clinicals studies could have reached their endpoints.

With the results from study 3 it can now be concluded that the refined region-specific technique is highly suitable for the intranasal N2B delivery of complex proteins such as IgGs. Further, the importance of the antigen-specificity of the antibody as well as that of its Fc domain can be confirmed. In general, the understanding of mechanisms of IgG transport processes from the nasal olfactory mucosa to the brain are an important step along the way of developing a standardized delivery route for therapeutics. It is widely accepted that FcRn plays a pivotal role in antibody transport across mucosal tissues [42,70,71,72,73]. In the presented study, the involvement of FcRn in IgG transport through the olfactory mucosa in vivo in mice could be shown first. Earlier, the role of FcRn in ex vivo porcine mucosa models and in primary cells from porcine nasal mucosa [38,39] has been demonstrated. According to the literature, FcRn transcytosis of IgG in mucosal epithelial cells act as a mechanism for IgG delivery to the underlying tissue [74,75]. This may explain the intracellular pathways observed in Figure 5A–D with full IgGs binding to either FcRn or FCGRs while extracellular pathways were evident when applying a scFv (Figure 5E,F). It can be only assumed that the underlying mechanisms for this involve immune surveillance where antigens captured from mucosal IgGs are presented to immune cells. A clue for this possible function may be the earlier presented disappearance of IgGs in lymphoid follicles of the nasal mucosa [38]. Unpublished data of the present study indicated that IgGs may also be degraded in the endosomal system as previously shown in primary cells [39]. Thus, it can be speculated that low doses of intranasal IgGs were degraded for immunological reasons and entered the nasal mucosa via passive extracellular pathways after a saturation of the Fc receptor system. However, further experiments are necessary to investigate dose-effects relationships for region-specific intranasally delivered IgGs.

It is hoped that with the presented technique the field of intranasal delivery can be advanced, and can contribute to a better standardization of in vivo data.

## 5. Conclusions

This project successfully achieved the specific targeting of the olfactory, or the respiratory region, by using a catheter-based refined technique that was previously developed and validated with 3D-printed casts of the murine nasal cavity under 3R criteria [25]. It was further demonstrated that region-specific administration with a fluorophore (sodium fluorescein), small (insulin detemir) and large proteins (a CNS-specific murine IgG1 and scFv, and a non-CNS targeting isotope control mAb) resulted in different organ distributions as well as different peripheral and central bioactivities in vivo. For immunoglobulins, transport along the olfactory route to the CNS via neuronal connections and distribution within the CNS was observed. In summary, it was successfully demonstrated that region-specific intranasal administration via the olfactory region resulted in improved brain targeting and in reduced peripheral targeting in rodents.
